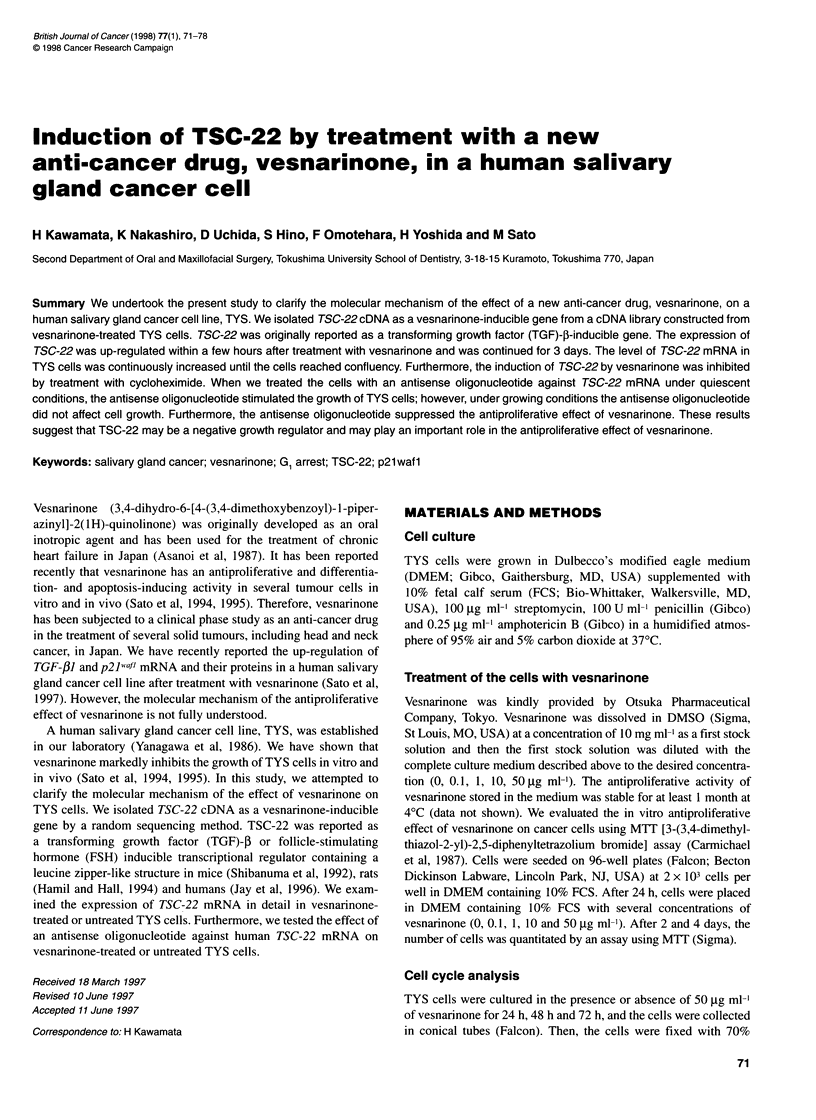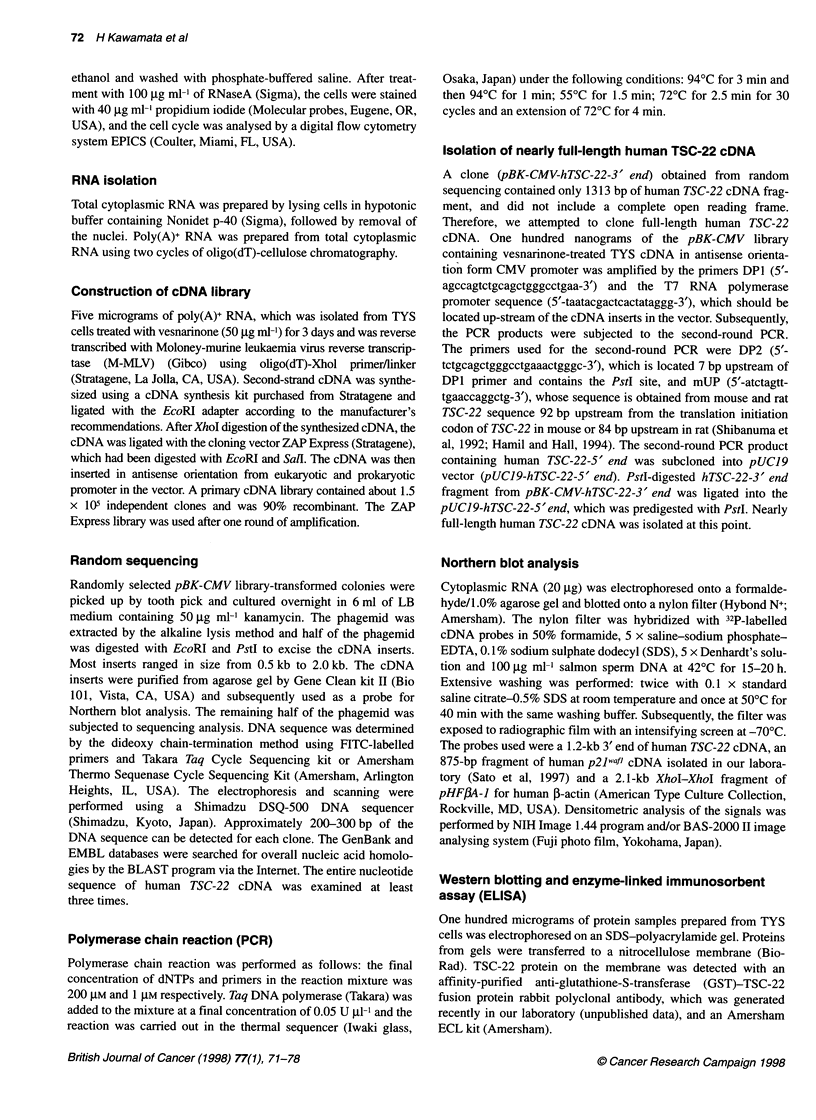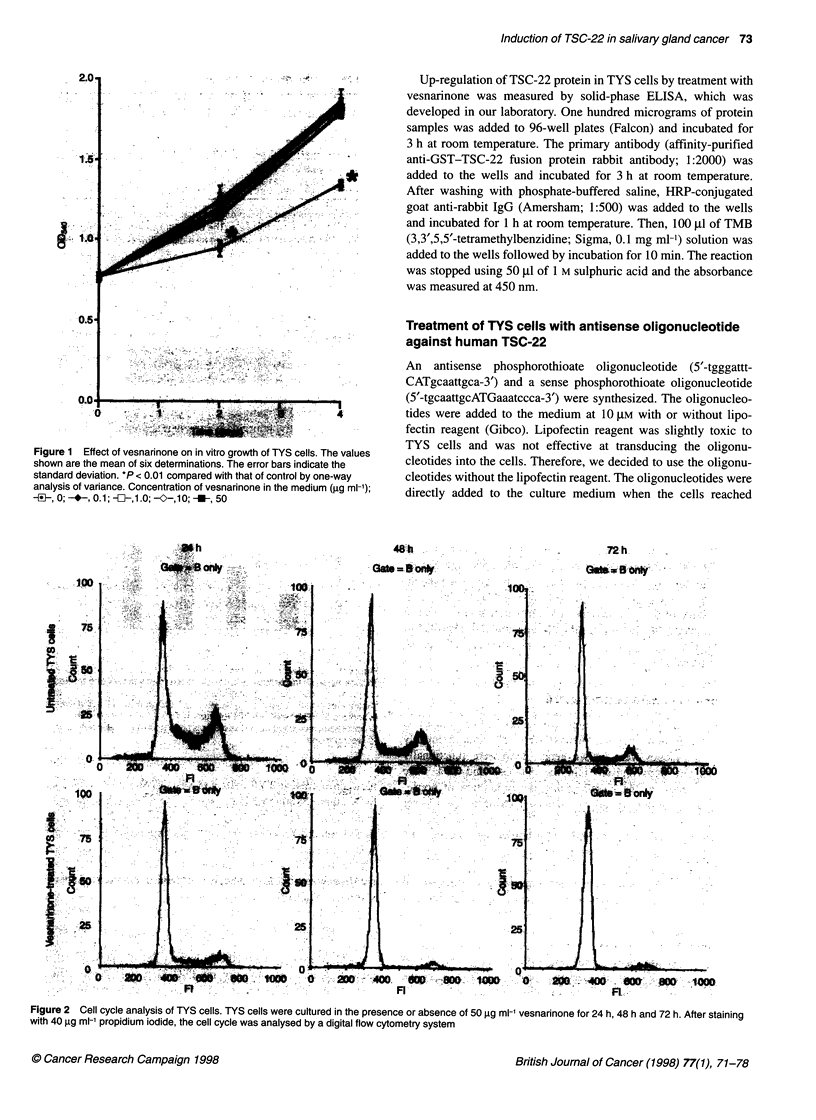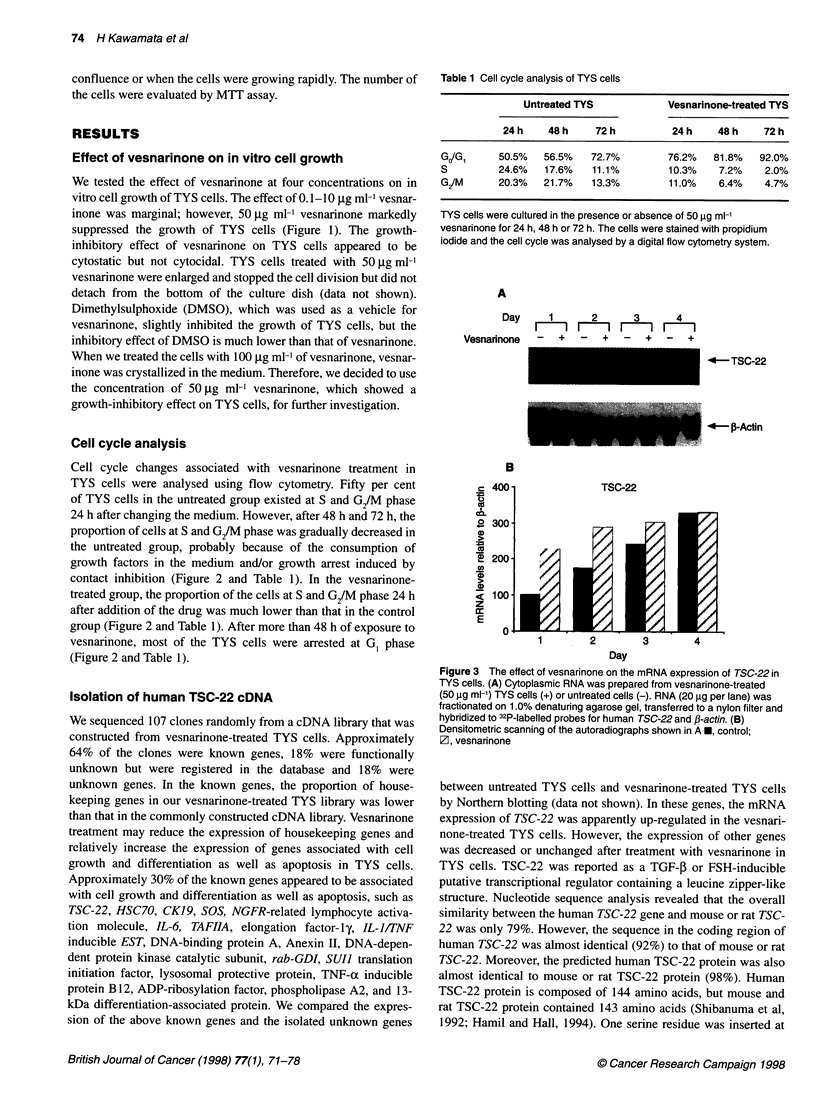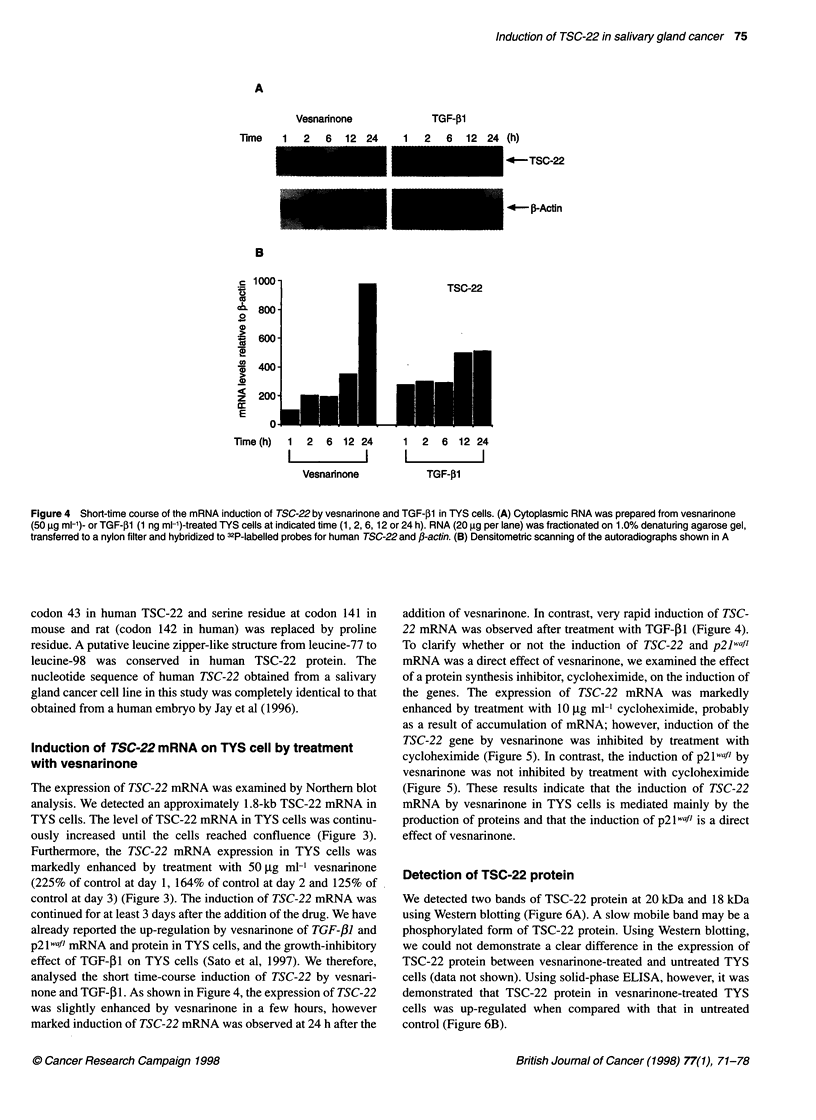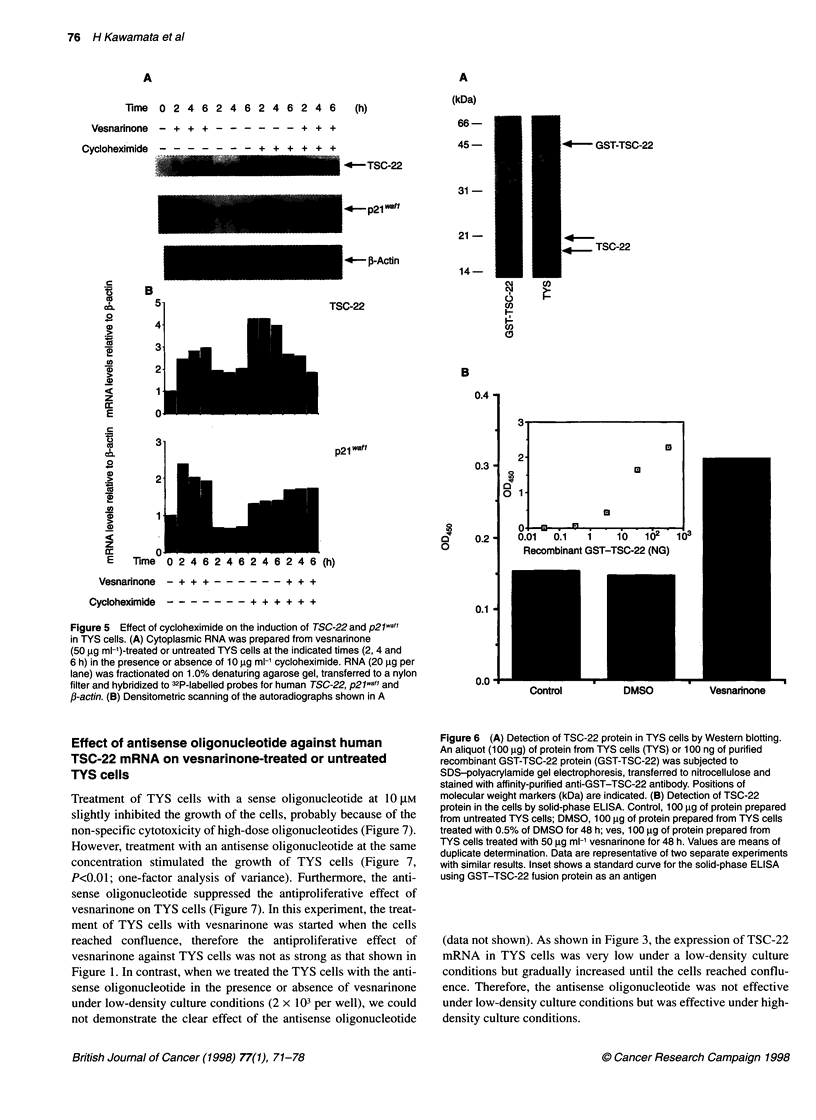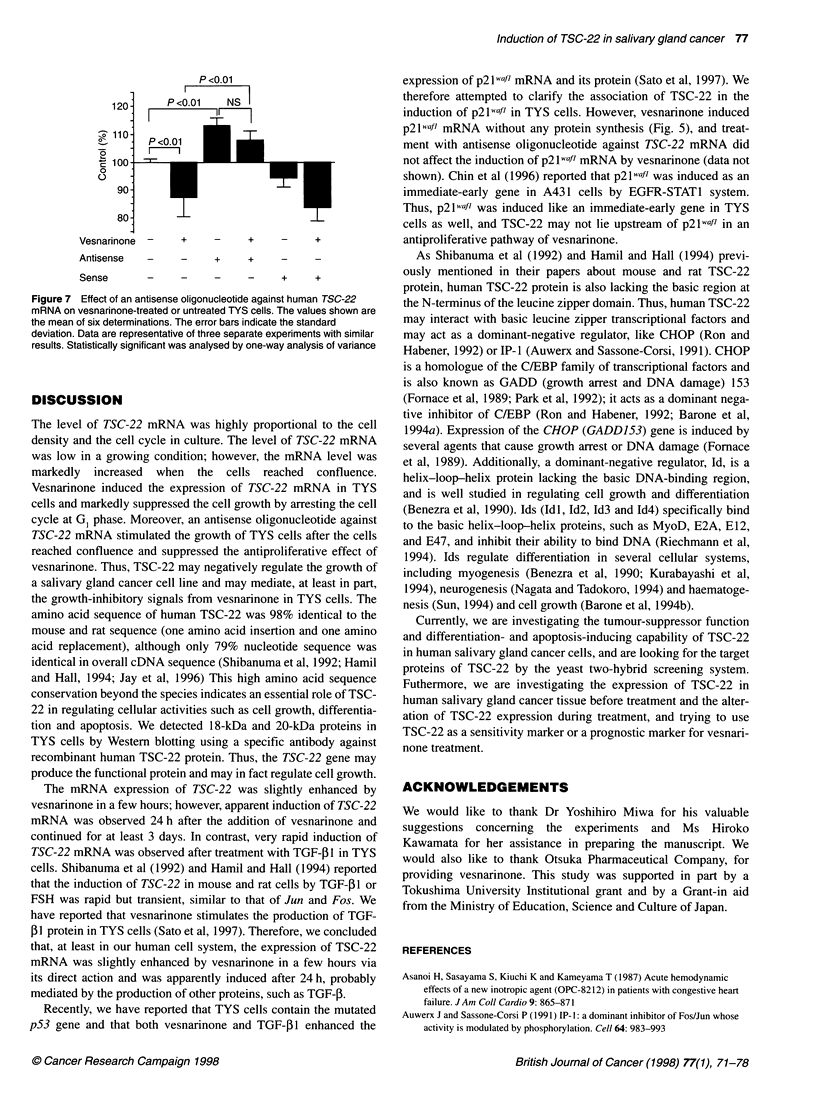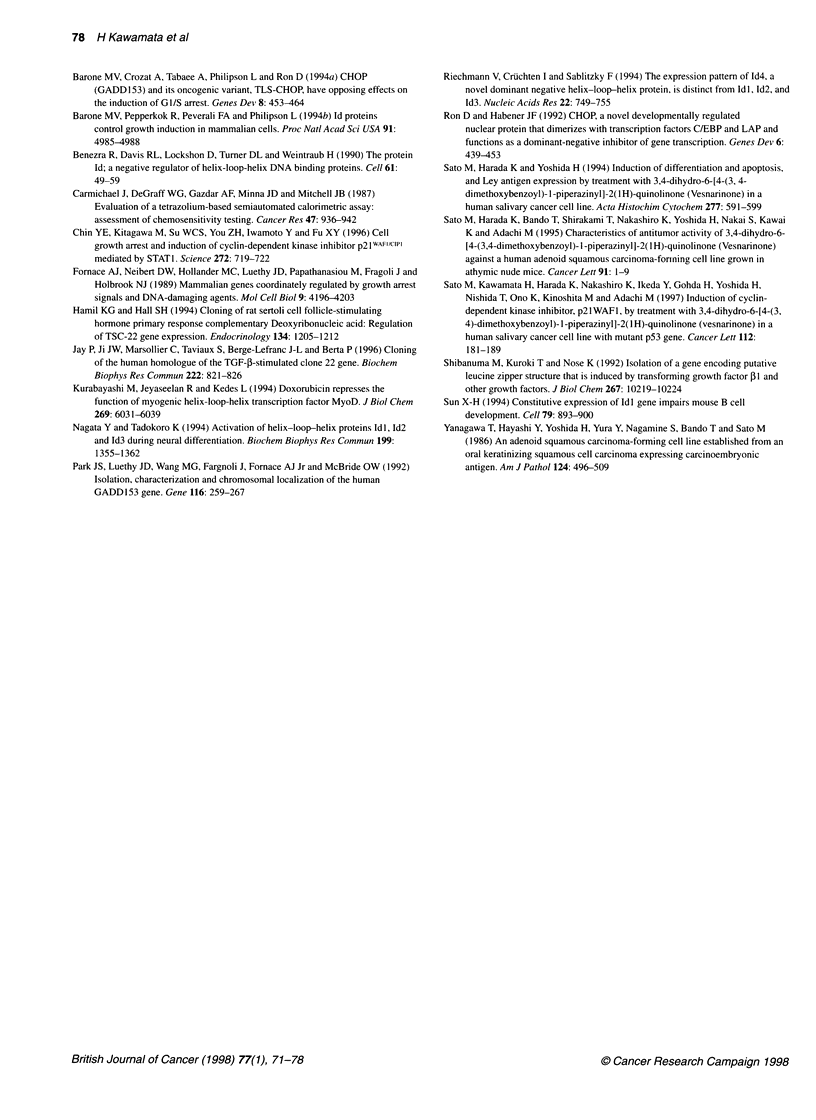# Induction of TSC-22 by treatment with a new anti-cancer drug, vesnarinone, in a human salivary gland cancer cell.

**DOI:** 10.1038/bjc.1998.11

**Published:** 1998

**Authors:** H. Kawamata, K. Nakashiro, D. Uchida, S. Hino, F. Omotehara, H. Yoshida, M. Sato

**Affiliations:** Second Department of Oral and Maxillofacial Surgery, Tokushima University School of Dentistry, Kuramoto, Japan.

## Abstract

**Images:**


British Journal of Cancer (1998) 77(1), 71–78
© 1998 Cancer Research Campaign

### Induction of TSC-22 by treatment with a new anti-cancer drug, vesnarinone, in a human salivary gland cancer cell

**H Kawamata, K Nakashiro, D Uchida, S Hino, F Omotehara, H Yoshida and M Sato**

Second Department of Oral and Maxillofacial Surgery, Tokushima University School of Dentistry, 3-18-15 Kuramoto, Tokushima 770, Japan

**Summary** We undertook the present study to clarify the molecular mechanism of the effect of a new anti-cancer drug, vesnarinone, on a human salivary gland cancer cell line, TYS. We isolated *TSC-22* cDNA as a vesnarinone-inducible gene from a cDNA library constructed from vesnarinone-treated TYS cells. *TSC-22* was originally reported as a transforming growth factor (TGF)-β-inducible gene. The expression of *TSC-22* was up-regulated within a few hours after treatment with vesnarinone and was continued for 3 days. The level of *TSC-22* mRNA in TYS cells was continuously increased until the cells reached confluency. Furthermore, the induction of *TSC-22* by vesnarinone was inhibited by treatment with cycloheximide. When we treated the cells with an antisense oligonucleotide against *TSC-22* mRNA under quiescent conditions, the antisense oligonucleotide stimulated the growth of TYS cells; however, under growing conditions the antisense oligonucleotide did not affect cell growth. Furthermore, the antisense oligonucleotide suppressed the antiproliferative effect of vesnarinone. These results suggest that TSC-22 may be a negative growth regulator and may play an important role in the antiproliferative effect of vesnarinone.

**Keywords:** salivary gland cancer; vesnarinone; $G_1$ arrest; TSC-22; p21waf1

Vesnarinone (3,4-dihydro-6-[4-(3,4-dimethoxybenzoyl)-1-piperazinyl]-2(1H)-quinolinone) was originally developed as an oral inotropic agent and has been used for the treatment of chronic heart failure in Japan (Asanoi et al, 1987). It has been reported recently that vesnarinone has an antiproliferative and differentiation- and apoptosis-inducing activity in several tumour cells in vitro and in vivo (Sato et al, 1994, 1995). Therefore, vesnarinone has been subjected to a clinical phase study as an anti-cancer drug in the treatment of several solid tumours, including head and neck cancer, in Japan. We have recently reported the up-regulation of *TGF-β1* and $p21^{waf1}$ mRNA and their proteins in a human salivary gland cancer cell line after treatment with vesnarinone (Sato et al, 1997). However, the molecular mechanism of the antiproliferative effect of vesnarinone is not fully understood.

A human salivary gland cancer cell line, TYS, was established in our laboratory (Yanagawa et al, 1986). We have shown that vesnarinone markedly inhibits the growth of TYS cells in vitro and in vivo (Sato et al, 1994, 1995). In this study, we attempted to clarify the molecular mechanism of the effect of vesnarinone on TYS cells. We isolated *TSC-22* cDNA as a vesnarinone-inducible gene by a random sequencing method. TSC-22 was reported as a transforming growth factor (TGF)-β or follicle-stimulating hormone (FSH) inducible transcriptional regulator containing a leucine zipper-like structure in mice (Shibanuma et al, 1992), rats (Hamil and Hall, 1994) and humans (Jay et al, 1996). We examined the expression of *TSC-22* mRNA in detail in vesnarinone-treated or untreated TYS cells. Furthermore, we tested the effect of an antisense oligonucleotide against human *TSC-22* mRNA on vesnarinone-treated or untreated TYS cells.

*Received 18 March 1997*
*Revised 10 June 1997*
*Accepted 11 June 1997*

*Correspondence to:* H Kawamata

### MATERIALS AND METHODS

### Cell culture

TYS cells were grown in Dulbecco's modified eagle medium (DMEM; Gibco, Gaithersburg, MD, USA) supplemented with 10% fetal calf serum (FCS; Bio-Whittaker, Walkersville, MD, USA), 100 μg $ml^{-1}$ streptomycin, 100 U $ml^{-1}$ penicillin (Gibco) and 0.25 μg $ml^{-1}$ amphotericin B (Gibco) in a humidified atmosphere of 95% air and 5% carbon dioxide at 37°C.

### Treatment of the cells with vesnarinone

Vesnarinone was kindly provided by Otsuka Pharmaceutical Company, Tokyo. Vesnarinone was dissolved in DMSO (Sigma, St Louis, MO, USA) at a concentration of 10 mg $ml^{-1}$ as a first stock solution and then the first stock solution was diluted with the complete culture medium described above to the desired concentration (0, 0.1, 1, 10, 50 μg $ml^{-1}$). The antiproliferative activity of vesnarinone stored in the medium was stable for at least 1 month at 4°C (data not shown). We evaluated the in vitro antiproliferative effect of vesnarinone on cancer cells using MTT [3-(3,4-dimethylthiazol-2-yl)-2,5-diphenyltetrazolium bromide] assay (Carmichael et al, 1987). Cells were seeded on 96-well plates (Falcon; Becton Dickinson Labware, Lincoln Park, NJ, USA) at $2 \times 10^3$ cells per well in DMEM containing 10% FCS. After 24 h, cells were placed in DMEM containing 10% FCS with several concentrations of vesnarinone (0, 0.1, 1, 10 and 50 μg $ml^{-1}$). After 2 and 4 days, the number of cells was quantitated by an assay using MTT (Sigma).

### Cell cycle analysis

TYS cells were cultured in the presence or absence of 50 μg $ml^{-1}$ of vesnarinone for 24 h, 48 h and 72 h, and the cells were collected in conical tubes (Falcon). Then, the cells were fixed with 70%

ethanol and washed with phosphate-buffered saline. After treatment with 100 μg ml$^{-1}$ of RNaseA (Sigma), the cells were stained with 40 μg ml$^{-1}$ propidium iodide (Molecular probes, Eugene, OR, USA), and the cell cycle was analysed by a digital flow cytometry system EPICS (Coulter, Miami, FL, USA).

### RNA isolation

Total cytoplasmic RNA was prepared by lysing cells in hypotonic buffer containing Nonidet p-40 (Sigma), followed by removal of the nuclei. Poly(A)$^+$ RNA was prepared from total cytoplasmic RNA using two cycles of oligo(dT)-cellulose chromatography.

### Construction of cDNA library

Five micrograms of poly(A)$^+$ RNA, which was isolated from TYS cells treated with vesnarinone (50 μg ml$^{-1}$) for 3 days and was reverse transcribed with Moloney-murine leukaemia virus reverse transcriptase (M-MLV) (Gibco) using oligo(dT)-XhoI primer/linker (Stratagene, La Jolla, CA, USA). Second-strand cDNA was synthesized using a cDNA synthesis kit purchased from Stratagene and ligated with the *Eco*RI adapter according to the manufacturer's recommendations. After *Xho*I digestion of the synthesized cDNA, the cDNA was ligated with the cloning vector ZAP Express (Stratagene), which had been digested with *Eco*RI and *Sal*I. The cDNA was then inserted in antisense orientation from eukaryotic and prokaryotic promoter in the vector. A primary cDNA library contained about 1.5 × 10$^5$ independent clones and was 90% recombinant. The ZAP Express library was used after one round of amplification.

### Random sequencing

Randomly selected *pBK-CMV* library-transformed colonies were picked up by tooth pick and cultured overnight in 6 ml of LB medium containing 50 μg ml$^{-1}$ kanamycin. The phagemid was extracted by the alkaline lysis method and half of the phagemid was digested with *Eco*RI and *Pst*I to excise the cDNA inserts. Most inserts ranged in size from 0.5 kb to 2.0 kb. The cDNA inserts were purified from agarose gel by Gene Clean kit II (Bio 101, Vista, CA, USA) and subsequently used as a probe for Northern blot analysis. The remaining half of the phagemid was subjected to sequencing analysis. DNA sequence was determined by the dideoxy chain-termination method using FITC-labelled primers and Takara *Taq* Cycle Sequencing kit or Amersham Thermo Sequenase Cycle Sequencing Kit (Amersham, Arlington Heights, IL, USA). The electrophoresis and scanning were performed using a Shimadzu DSQ-500 DNA sequencer (Shimadzu, Kyoto, Japan). Approximately 200–300 bp of the DNA sequence can be detected for each clone. The GenBank and EMBL databases were searched for overall nucleic acid homologies by the BLAST program via the Internet. The entire nucleotide sequence of human *TSC-22* cDNA was examined at least three times.

### Polymerase chain reaction (PCR)

Polymerase chain reaction was performed as follows: the final concentration of dNTPs and primers in the reaction mixture was 200 μM and 1 μM respectively. *Taq* DNA polymerase (Takara) was added to the mixture at a final concentration of 0.05 U μl$^{-1}$ and the reaction was carried out in the thermal sequencer (Iwaki glass, Osaka, Japan) under the following conditions: 94°C for 3 min and then 94°C for 1 min; 55°C for 1.5 min; 72°C for 2.5 min for 30 cycles and an extension of 72°C for 4 min.

### Isolation of nearly full-length human TSC-22 cDNA

A clone (*pBK-CMV-hTSC-22-3′ end*) obtained from random sequencing contained only 1313 bp of human *TSC-22* cDNA fragment, and did not include a complete open reading frame. Therefore, we attempted to clone full-length human *TSC-22* cDNA. One hundred nanograms of the *pBK-CMV* library containing vesnarinone-treated TYS cDNA in antisense orientation form CMV promoter was amplified by the primers DP1 (5′-agccagtctgcagctgggcctgaa-3′) and the T7 RNA polymerase promoter sequence (5′-taatacgactcactatagg g-3′), which should be located up-stream of the cDNA inserts in the vector. Subsequently, the PCR products were subjected to the second-round PCR. The primers used for the second-round PCR were DP2 (5′-tctgcagctgggcctgaaactgggc-3′), which is located 7 bp upstream of DP1 primer and contains the *Pst*I site, and mUP (5′-atctagtt-tgaaccaggctg-3′), whose sequence is obtained from mouse and rat *TSC-22* sequence 92 bp upstream from the translation initiation codon of *TSC-22* in mouse or 84 bp upstream in rat (Shibanuma et al, 1992; Hamil and Hall, 1994). The second-round PCR product containing human *TSC-22-5′ end* was subcloned into *pUC19* vector (*pUC19-hTSC-22-5′ end*). *Pst*I-digested *hTSC-22-3′ end* fragment from *pBK-CMV-hTSC-22-3′ end* was ligated into the *pUC19-hTSC-22-5′ end*, which was predigested with *Pst*I. Nearly full-length human *TSC-22* cDNA was isolated at this point.

### Northern blot analysis

Cytoplasmic RNA (20 μg) was electrophoresed onto a formaldehyde/1.0% agarose gel and blotted onto a nylon filter (Hybond N$^+$; Amersham). The nylon filter was hybridized with $^{32}$P-labelled cDNA probes in 50% formamide, 5 × saline–sodium phosphate–EDTA, 0.1% sodium sulphate dodecyl (SDS), 5 × Denhardt's solution and 100 μg ml$^{-1}$ salmon sperm DNA at 42°C for 15–20 h. Extensive washing was performed: twice with 0.1 × standard saline citrate–0.5% SDS at room temperature and once at 50°C for 40 min with the same washing buffer. Subsequently, the filter was exposed to radiographic film with an intensifying screen at –70°C. The probes used were a 1.2-kb 3′ end of human *TSC-22* cDNA, an 875-bp fragment of human $p21^{waf1}$ cDNA isolated in our laboratory (Sato et al, 1997) and a 2.1-kb *Xho*I–*Xho*I fragment of *pHFβA-1* for human β-actin (American Type Culture Collection, Rockville, MD, USA). Densitometric analysis of the signals was performed by NIH Image 1.44 program and/or BAS-2000 II image analysing system (Fuji photo film, Yokohama, Japan).

### Western blotting and enzyme-linked immunosorbent assay (ELISA)

One hundred micrograms of protein samples prepared from TYS cells was electrophoresed on an SDS–polyacrylamide gel. Proteins from gels were transferred to a nitrocellulose membrane (Bio-Rad). TSC-22 protein on the membrane was detected with an affinity-purified anti-glutathione-S-transferase (GST)–TSC-22 fusion protein rabbit polyclonal antibody, which was generated recently in our laboratory (unpublished data), and an Amersham ECL kit (Amersham).

© Cancer Research Campaign 1998

**Figure 1** Effect of vesnarinone on in vitro growth of TYS cells. The values shown are the mean of six determinations. The error bars indicate the standard deviation. *$P < 0.01$ compared with that of control by one-way analysis of variance. Concentration of vesnarinone in the medium (µg $ml^{-1}$); -⊡-, 0; -◆-, 0.1; -□-,1.0; -◇-,10; -■-, 50

Up-regulation of TSC-22 protein in TYS cells by treatment with vesnarinone was measured by solid-phase ELISA, which was developed in our laboratory. One hundred micrograms of protein samples was added to 96-well plates (Falcon) and incubated for 3 h at room temperature. The primary antibody (affinity-purified anti-GST–TSC-22 fusion protein rabbit antibody; 1:2000) was added to the wells and incubated for 3 h at room temperature. After washing with phosphate-buffered saline, HRP-conjugated goat anti-rabbit IgG (Amersham; 1:500) was added to the wells and incubated for 1 h at room temperature. Then, 100 µl of TMB (3,3′,5,5′-tetramethylbenzidine; Sigma, 0.1 mg $ml^{-1}$) solution was added to the wells followed by incubation for 10 min. The reaction was stopped using 50 µl of 1 M sulphuric acid and the absorbance was measured at 450 nm.

### Treatment of TYS cells with antisense oligonucleotide against human TSC-22

An antisense phosphorothioate oligonucleotide (5′-tgggatttCATgcaattgca-3′) and a sense phosphorothioate oligonucleotide (5′-tgcaattgcATGaaatccca-3′) were synthesized. The oligonucleotides were added to the medium at 10 µM with or without lipofectin reagent (Gibco). Lipofectin reagent was slightly toxic to TYS cells and was not effective at transducing the oligonucleotides into the cells. Therefore, we decided to use the oligonucleotides without the lipofectin reagent. The oligonucleotides were directly added to the culture medium when the cells reached

**Figure 2** Cell cycle analysis of TYS cells. TYS cells were cultured in the presence or absence of 50 µg $ml^{-1}$ vesnarinone for 24 h, 48 h and 72 h. After staining with 40 µg $ml^{-1}$ propidium iodide, the cell cycle was analysed by a digital flow cytometry system

© Cancer Research Campaign 1998

confluence or when the cells were growing rapidly. The number of the cells were evaluated by MTT assay.

### RESULTS

### Effect of vesnarinone on in vitro cell growth

We tested the effect of vesnarinone at four concentrations on in vitro cell growth of TYS cells. The effect of 0.1–10 μg ml$^{-1}$ vesnarinone was marginal; however, 50 μg ml$^{-1}$ vesnarinone markedly suppressed the growth of TYS cells (Figure 1). The growth-inhibitory effect of vesnarinone on TYS cells appeared to be cytostatic but not cytocidal. TYS cells treated with 50 μg ml$^{-1}$ vesnarinone were enlarged and stopped the cell division but did not detach from the bottom of the culture dish (data not shown). Dimethylsulphoxide (DMSO), which was used as a vehicle for vesnarinone, slightly inhibited the growth of TYS cells, but the inhibitory effect of DMSO is much lower than that of vesnarinone. When we treated the cells with 100 μg ml$^{-1}$ of vesnarinone, vesnarinone was crystallized in the medium. Therefore, we decided to use the concentration of 50 μg ml$^{-1}$ vesnarinone, which showed a growth-inhibitory effect on TYS cells, for further investigation.

### Cell cycle analysis

Cell cycle changes associated with vesnarinone treatment in TYS cells were analysed using flow cytometry. Fifty per cent of TYS cells in the untreated group existed at S and $G_2$/M phase 24 h after changing the medium. However, after 48 h and 72 h, the proportion of cells at S and $G_2$/M phase was gradually decreased in the untreated group, probably because of the consumption of growth factors in the medium and/or growth arrest induced by contact inhibition (Figure 2 and Table 1). In the vesnarinone-treated group, the proportion of the cells at S and $G_2$/M phase 24 h after addition of the drug was much lower than that in the control group (Figure 2 and Table 1). After more than 48 h of exposure to vesnarinone, most of the TYS cells were arrested at $G_1$ phase (Figure 2 and Table 1).

### Isolation of human TSC-22 cDNA

We sequenced 107 clones randomly from a cDNA library that was constructed from vesnarinone-treated TYS cells. Approximately 64% of the clones were known genes, 18% were functionally unknown but were registered in the database and 18% were unknown genes. In the known genes, the proportion of housekeeping genes in our vesnarinone-treated TYS library was lower than that in the commonly constructed cDNA library. Vesnarinone treatment may reduce the expression of housekeeping genes and relatively increase the expression of genes associated with cell growth and differentiation as well as apoptosis in TYS cells. Approximately 30% of the known genes appeared to be associated with cell growth and differentiation as well as apoptosis, such as *TSC-22*, *HSC70*, *CK19*, *SOS*, *NGFR*-related lymphocyte activation molecule, *IL-6*, *TAFIIA*, elongation factor-1γ, *IL-1/TNF* inducible *EST*, DNA-binding protein A, Anexin II, DNA-dependent protein kinase catalytic subunit, *rab-GDI*, *SUI1* translation initiation factor, lysosomal protective protein, TNF-α inducible protein B12, ADP-ribosylation factor, phospholipase A2, and 13-kDa differentiation-associated protein. We compared the expression of the above known genes and the isolated unknown genes between untreated TYS cells and vesnarinone-treated TYS cells by Northern blotting (data not shown). In these genes, the mRNA expression of *TSC-22* was apparently up-regulated in the vesnarinone-treated TYS cells. However, the expression of other genes was decreased or unchanged after treatment with vesnarinone in TYS cells. TSC-22 was reported as a TGF-β or FSH-inducible putative transcriptional regulator containing a leucine zipper-like structure. Nucleotide sequence analysis revealed that the overall similarity between the human *TSC-22* gene and mouse or rat *TSC-22* was only 79%. However, the sequence in the coding region of human *TSC-22* was almost identical (92%) to that of mouse or rat *TSC-22*. Moreover, the predicted human TSC-22 protein was also almost identical to mouse or rat TSC-22 protein (98%). Human TSC-22 protein is composed of 144 amino acids, but mouse and rat TSC-22 protein contained 143 amino acids (Shibanuma et al, 1992; Hamil and Hall, 1994). One serine residue was inserted at

**Table 1** Cell cycle analysis of TYS cells

| | Untreated TYS | | | Vesnarinone-treated TYS | | |
|---|---|---|---|---|---|---|
| | 24 h | 48 h | 72 h | 24 h | 48 h | 72 h |
| $G_0/G_1$ | 50.5% | 56.5% | 72.7% | 76.2% | 81.8% | 92.0% |
| S | 24.6% | 17.6% | 11.1% | 10.3% | 7.2% | 2.0% |
| $G_2$/M | 20.3% | 21.7% | 13.3% | 11.0% | 6.4% | 4.7% |

TYS cells were cultured in the presence or absence of 50 μg ml$^{-1}$ vesnarinone for 24 h, 48 h or 72 h. The cells were stained with propidium iodide and the cell cycle was analysed by a digital flow cytometry system.

**Figure 3** The effect of vesnarinone on the mRNA expression of *TSC-22* in TYS cells. (**A**) Cytoplasmic RNA was prepared from vesnarinone-treated (50 μg ml$^{-1}$) TYS cells (+) or untreated cells (–). RNA (20 μg per lane) was fractionated on 1.0% denaturing agarose gel, transferred to a nylon filter and hybridized to $^{32}$P-labelled probes for human *TSC-22* and *β-actin*. (**B**) Densitometric scanning of the autoradiographs shown in A ■, control; ▨, vesnarinone

© Cancer Research Campaign 1998

**Figure 4** Short-time course of the mRNA induction of *TSC-22* by vesnarinone and TGF-β1 in TYS cells. (**A**) Cytoplasmic RNA was prepared from vesnarinone (50 μg $ml^{-1}$)- or TGF-β1 (1 ng $ml^{-1}$)-treated TYS cells at indicated time (1, 2, 6, 12 or 24 h). RNA (20 μg per lane) was fractionated on 1.0% denaturing agarose gel, transferred to a nylon filter and hybridized to $^{32}$P-labelled probes for human *TSC-22* and *β-actin*. (**B**) Densitometric scanning of the autoradiographs shown in A

codon 43 in human TSC-22 and serine residue at codon 141 in mouse and rat (codon 142 in human) was replaced by proline residue. A putative leucine zipper-like structure from leucine-77 to leucine-98 was conserved in human TSC-22 protein. The nucleotide sequence of human *TSC-22* obtained from a salivary gland cancer cell line in this study was completely identical to that obtained from a human embryo by Jay et al (1996).

### Induction of *TSC-22* mRNA on TYS cell by treatment with vesnarinone

The expression of *TSC-22* mRNA was examined by Northern blot analysis. We detected an approximately 1.8-kb TSC-22 mRNA in TYS cells. The level of TSC-22 mRNA in TYS cells was continuously increased until the cells reached confluence (Figure 3). Furthermore, the *TSC-22* mRNA expression in TYS cells was markedly enhanced by treatment with 50 μg $ml^{-1}$ vesnarinone (225% of control at day 1, 164% of control at day 2 and 125% of control at day 3) (Figure 3). The induction of *TSC-22* mRNA was continued for at least 3 days after the addition of the drug. We have already reported the up-regulation by vesnarinone of *TGF-β1* and p21$^{waf1}$ mRNA and protein in TYS cells, and the growth-inhibitory effect of TGF-β1 on TYS cells (Sato et al, 1997). We therefore, analysed the short time-course induction of *TSC-22* by vesnarinone and TGF-β1. As shown in Figure 4, the expression of *TSC-22* was slightly enhanced by vesnarinone in a few hours, however marked induction of *TSC-22* mRNA was observed at 24 h after the addition of vesnarinone. In contrast, very rapid induction of *TSC-22* mRNA was observed after treatment with TGF-β1 (Figure 4). To clarify whether or not the induction of *TSC-22* and *p21$^{waf1}$* mRNA was a direct effect of vesnarinone, we examined the effect of a protein synthesis inhibitor, cycloheximide, on the induction of the genes. The expression of *TSC-22* mRNA was markedly enhanced by treatment with 10 μg $ml^{-1}$ cycloheximide, probably as a result of accumulation of mRNA; however, induction of the *TSC-22* gene by vesnarinone was inhibited by treatment with cycloheximide (Figure 5). In contrast, the induction of p21$^{waf1}$ by vesnarinone was not inhibited by treatment with cycloheximide (Figure 5). These results indicate that the induction of *TSC-22* mRNA by vesnarinone in TYS cells is mediated mainly by the production of proteins and that the induction of p21$^{waf1}$ is a direct effect of vesnarinone.

### Detection of TSC-22 protein

We detected two bands of TSC-22 protein at 20 kDa and 18 kDa using Western blotting (Figure 6A). A slow mobile band may be a phosphorylated form of TSC-22 protein. Using Western blotting, we could not demonstrate a clear difference in the expression of TSC-22 protein between vesnarinone-treated and untreated TYS cells (data not shown). Using solid-phase ELISA, however, it was demonstrated that TSC-22 protein in vesnarinone-treated TYS cells was up-regulated when compared with that in untreated control (Figure 6B).

© Cancer Research Campaign 1998

**Figure 5** Effect of cycloheximide on the induction of *TSC-22* and *p21^waf1^* in TYS cells. (**A**) Cytoplasmic RNA was prepared from vesnarinone (50 μg ml$^{-1}$)-treated or untreated TYS cells at the indicated times (2, 4 and 6 h) in the presence or absence of 10 μg ml$^{-1}$ cycloheximide. RNA (20 μg per lane) was fractionated on 1.0% denaturing agarose gel, transferred to a nylon filter and hybridized to $^{32}$P-labelled probes for human *TSC-22, p21$^{waf1}$* and *β-actin.* (**B**) Densitometric scanning of the autoradiographs shown in A

**Figure 6** (**A**) Detection of TSC-22 protein in TYS cells by Western blotting. An aliquot (100 μg) of protein from TYS cells (TYS) or 100 ng of purified recombinant GST-TSC-22 protein (GST-TSC-22) was subjected to SDS–polyacrylamide gel electrophoresis, transferred to nitrocellulose and stained with affinity-purified anti-GST–TSC-22 antibody. Positions of molecular weight markers (kDa) are indicated. (**B**) Detection of TSC-22 protein in the cells by solid-phase ELISA. Control, 100 μg of protein prepared from untreated TYS cells; DMSO, 100 μg of protein prepared from TYS cells treated with 0.5% of DMSO for 48 h; ves, 100 μg of protein prepared from TYS cells treated with 50 μg ml$^{-1}$ vesnarinone for 48 h. Values are means of duplicate determination. Data are representative of two separate experiments with similar results. Inset shows a standard curve for the solid-phase ELISA using GST–TSC-22 fusion protein as an antigen

### Effect of antisense oligonucleotide against human TSC-22 mRNA on vesnarinone-treated or untreated TYS cells

Treatment of TYS cells with a sense oligonucleotide at 10 μM slightly inhibited the growth of the cells, probably because of the non-specific cytotoxicity of high-dose oligonucleotides (Figure 7). However, treatment with an antisense oligonucleotide at the same concentration stimulated the growth of TYS cells (Figure 7, $P<0.01$; one-factor analysis of variance). Furthermore, the antisense oligonucleotide suppressed the antiproliferative effect of vesnarinone on TYS cells (Figure 7). In this experiment, the treatment of TYS cells with vesnarinone was started when the cells reached confluence, therefore the antiproliferative effect of vesnarinone against TYS cells was not as strong as that shown in Figure 1. In contrast, when we treated the TYS cells with the antisense oligonucleotide in the presence or absence of vesnarinone under low-density culture conditions ($2 \times 10^3$ per well), we could not demonstrate the clear effect of the antisense oligonucleotide (data not shown). As shown in Figure 3, the expression of TSC-22 mRNA in TYS cells was very low under a low-density culture conditions but gradually increased until the cells reached confluence. Therefore, the antisense oligonucleotide was not effective under low-density culture conditions but was effective under high-density culture conditions.

© Cancer Research Campaign 1998

**Figure 7** Effect of an antisense oligonucleotide against human *TSC-22* mRNA on vesnarinone-treated or untreated TYS cells. The values shown are the mean of six determinations. The error bars indicate the standard deviation. Data are representative of three separate experiments with similar results. Statistically significant was analysed by one-way analysis of variance

### DISCUSSION

The level of *TSC-22* mRNA was highly proportional to the cell density and the cell cycle in culture. The level of *TSC-22* mRNA was low in a growing condition; however, the mRNA level was markedly increased when the cells reached confluence. Vesnarinone induced the expression of *TSC-22* mRNA in TYS cells and markedly suppressed the cell growth by arresting the cell cycle at $G_1$ phase. Moreover, an antisense oligonucleotide against *TSC-22* mRNA stimulated the growth of TYS cells after the cells reached confluence and suppressed the antiproliferative effect of vesnarinone. Thus, TSC-22 may negatively regulate the growth of a salivary gland cancer cell line and may mediate, at least in part, the growth-inhibitory signals from vesnarinone in TYS cells. The amino acid sequence of human TSC-22 was 98% identical to the mouse and rat sequence (one amino acid insertion and one amino acid replacement), although only 79% nucleotide sequence was identical in overall cDNA sequence (Shibanuma et al, 1992; Hamil and Hall, 1994; Jay et al, 1996) This high amino acid sequence conservation beyond the species indicates an essential role of TSC-22 in regulating cellular activities such as cell growth, differentiation and apoptosis. We detected 18-kDa and 20-kDa proteins in TYS cells by Western blotting using a specific antibody against recombinant human TSC-22 protein. Thus, the *TSC-22* gene may produce the functional protein and may in fact regulate cell growth.

The mRNA expression of *TSC-22* was slightly enhanced by vesnarinone in a few hours; however, apparent induction of *TSC-22* mRNA was observed 24 h after the addition of vesnarinone and continued for at least 3 days. In contrast, very rapid induction of *TSC-22* mRNA was observed after treatment with TGF-β1 in TYS cells. Shibanuma et al (1992) and Hamil and Hall (1994) reported that the induction of *TSC-22* in mouse and rat cells by TGF-β1 or FSH was rapid but transient, similar to that of *Jun* and *Fos*. We have reported that vesnarinone stimulates the production of TGF-β1 protein in TYS cells (Sato et al, 1997). Therefore, we concluded that, at least in our human cell system, the expression of TSC-22 mRNA was slightly enhanced by vesnarinone in a few hours via its direct action and was apparently induced after 24 h, probably mediated by the production of other proteins, such as TGF-β.

Recently, we have reported that TYS cells contain the mutated *p53* gene and that both vesnarinone and TGF-β1 enhanced the expression of $p21^{waf1}$ mRNA and its protein (Sato et al, 1997). We therefore attempted to clarify the association of TSC-22 in the induction of $p21^{waf1}$ in TYS cells. However, vesnarinone induced $p21^{waf1}$ mRNA without any protein synthesis (Fig. 5), and treatment with antisense oligonucleotide against *TSC-22* mRNA did not affect the induction of $p21^{waf1}$ mRNA by vesnarinone (data not shown). Chin et al (1996) reported that $p21^{waf1}$ was induced as an immediate-early gene in A431 cells by EGFR-STAT1 system. Thus, $p21^{waf1}$ was induced like an immediate-early gene in TYS cells as well, and TSC-22 may not lie upstream of $p21^{waf1}$ in an antiproliferative pathway of vesnarinone.

As Shibanuma et al (1992) and Hamil and Hall (1994) previously mentioned in their papers about mouse and rat TSC-22 protein, human TSC-22 protein is also lacking the basic region at the N-terminus of the leucine zipper domain. Thus, human TSC-22 may interact with basic leucine zipper transcriptional factors and may act as a dominant-negative regulator, like CHOP (Ron and Habener, 1992) or IP-1 (Auwerx and Sassone-Corsi, 1991). CHOP is a homologue of the C/EBP family of transcriptional factors and is also known as GADD (growth arrest and DNA damage) 153 (Fornace et al, 1989; Park et al, 1992); it acts as a dominant negative inhibitor of C/EBP (Ron and Habener, 1992; Barone et al, 1994*a*). Expression of the *CHOP* (*GADD153*) gene is induced by several agents that cause growth arrest or DNA damage (Fornace et al, 1989). Additionally, a dominant-negative regulator, Id, is a helix–loop–helix protein lacking the basic DNA-binding region, and is well studied in regulating cell growth and differentiation (Benezra et al, 1990). Ids (Id1, Id2, Id3 and Id4) specifically bind to the basic helix–loop–helix proteins, such as MyoD, E2A, E12, and E47, and inhibit their ability to bind DNA (Riechmann et al, 1994). Ids regulate differentiation in several cellular systems, including myogenesis (Benezra et al, 1990; Kurabayashi et al, 1994), neurogenesis (Nagata and Tadokoro, 1994) and haematogenesis (Sun, 1994) and cell growth (Barone et al, 1994b).

Currently, we are investigating the tumour-suppressor function and differentiation- and apoptosis-inducing capability of TSC-22 in human salivary gland cancer cells, and are looking for the target proteins of TSC-22 by the yeast two-hybrid screening system. Futhermore, we are investigating the expression of TSC-22 in human salivary gland cancer tissue before treatment and the alteration of TSC-22 expression during treatment, and trying to use TSC-22 as a sensitivity marker or a prognostic marker for vesnarinone treatment.

### ACKNOWLEDGEMENTS

We would like to thank Dr Yoshihiro Miwa for his valuable suggestions concerning the experiments and Ms Hiroko Kawamata for her assistance in preparing the manuscript. We would also like to thank Otsuka Pharmaceutical Company, for providing vesnarinone. This study was supported in part by a Tokushima University Institutional grant and by a Grant-in aid from the Ministry of Education, Science and Culture of Japan.

### REFERENCES

Asanoi H, Sasayama S, Kiuchi K and Kameyama T (1987) Acute hemodynamic effects of a new inotropic agent (OPC-8212) in patients with congestive heart failure. *J Am Coll Cardio* **9**: 865–871

Auwerx J and Sassone-Corsi P (1991) IP-1: a dominant inhibitor of Fos/Jun whose activity is modulated by phosphorylation. *Cell* **64**: 983–993

© Cancer Research Campaign 1998

Barone MV, Crozat A, Tabaee A, Philipson L and Ron D (1994*a*) CHOP (GADD153) and its oncogenic variant, TLS-CHOP, have opposing effects on the induction of G1/S arrest. *Genes Dev* **8**: 453–464

Barone MV, Pepperkok R, Peverali FA and Philipson L (1994*b*) Id proteins control growth induction in mammalian cells. *Proc Natl Acad Sci USA* **91**: 4985–4988

Benezra R, Davis RL, Lockshon D, Turner DL and Weintraub H (1990) The protein Id; a negative regulator of helix-loop-helix DNA binding proteins. *Cell* **61**: 49–59

Carmichael J, DeGraff WG, Gazdar AF, Minna JD and Mitchell JB (1987) Evaluation of a tetrazolium-based semiautomated calorimetric assay: assessment of chemosensitivity testing. *Cancer Res* **47**: 936–942

Chin YE, Kitagawa M, Su WCS, You ZH, Iwamoto Y and Fu XY (1996) Cell growth arrest and induction of cyclin-dependent kinase inhibitor $p21^{WAF1/CIP1}$ mediated by STAT1. *Science* **272**: 719–722

Fornace AJ, Neibert DW, Hollander MC, Luethy JD, Papathanasiou M, Fragoli J and Holbrook NJ (1989) Mammalian genes coordinately regulated by growth arrest signals and DNA-damaging agents. *Mol Cell Biol* **9**: 4196–4203

Hamil KG and Hall SH (1994) Cloning of rat sertoli cell follicle-stimulating hormone primary response complementary Deoxyribonucleic acid: Regulation of TSC-22 gene expression. *Endocrinology* **134**: 1205–1212

Jay P, Ji JW, Marsollier C, Taviaux S, Berge-Lefranc J-L and Berta P (1996) Cloning of the human homologue of the TGF-β-stimulated clone 22 gene. *Biochem Biophys Res Commun* **222**: 821–826

Kurabayashi M, Jeyaseelan R and Kedes L (1994) Doxorubicin represses the function of myogenic helix-loop-helix transcription factor MyoD. *J Biol Chem* **269**: 6031–6039

Nagata Y and Tadokoro K (1994) Activation of helix–loop–helix proteins Id1, Id2 and Id3 during neural differentiation. *Biochem Biophys Res Commun* **199**: 1355–1362

Park JS, Luethy JD, Wang MG, Fargnoli J, Fornace AJ Jr and McBride OW (1992) Isolation, characterization and chromosomal localization of the human GADD153 gene. *Gene* **116**: 259–267

Riechmann V, Crüchten I and Sablitzky F (1994) The expression pattern of Id4, a novel dominant negative helix–loop–helix protein, is distinct from Id1, Id2, and Id3. *Nucleic Acids Res* **22**: 749–755

Ron D and Habener JF (1992) CHOP, a novel developmentally regulated nuclear protein that dimerizes with transcription factors C/EBP and LAP and functions as a dominant-negative inhibitor of gene transcription. *Genes Dev* **6**: 439–453

Sato M, Harada K and Yoshida H (1994) Induction of differentiation and apoptosis, and Ley antigen expression by treatment with 3,4-dihydro-6-[4-(3, 4-dimethoxybenzoyl)-1-piperazinyl]-2(1H)-quinolinone (Vesnarinone) in a human salivary cancer cell line. *Acta Histochim Cytochem* **277**: 591–599

Sato M, Harada K, Bando T, Shirakami T, Nakashiro K, Yoshida H, Nakai S, Kawai K and Adachi M (1995) Characteristics of antitumor activity of 3,4-dihydro-6-[4-(3,4-dimethoxybenzoyl)-1-piperazinyl]-2(1H)-quinolinone (Vesnarinone) against a human adenoid squamous carcinoma-forming cell line grown in athymic nude mice. *Cancer Lett* **91**: 1–9

Sato M, Kawamata H, Harada K, Nakashiro K, Ikeda Y, Gohda H, Yoshida H, Nishida T, Ono K, Kinoshita M and Adachi M (1997) Induction of cyclin-dependent kinase inhibitor, p21WAF1, by treatment with 3,4-dihydro-6-[4-(3, 4)-dimethoxybenzoyl)-1-piperazinyl]-2(1H)-quinolinone (vesnarinone) in a human salivary cancer cell line with mutant p53 gene. *Cancer Lett* **112**: 181–189

Shibanuma M, Kuroki T and Nose K (1992) Isolation of a gene encoding putative leucine zipper structure that is induced by transforming growth factor β1 and other growth factors. *J Biol Chem* **267**: 10219–10224

Sun X-H (1994) Constitutive expression of Id1 gene impairs mouse B cell development. *Cell* **79**: 893–900

Yanagawa T, Hayashi Y, Yoshida H, Yura Y, Nagamine S, Bando T and Sato M (1986) An adenoid squamous carcinoma-forming cell line established from an oral keratinizing squamous cell carcinoma expressing carcinoembryonic antigen. *Am J Pathol* **124**: 496–509

© Cancer Research Campaign 1998